# AI-Enabled Medical Education: Threads of Change, Promising Futures, and Risky Realities Across Four Potential Future Worlds

**DOI:** 10.2196/50373

**Published:** 2023-12-25

**Authors:** Michelle I Knopp, Eric J Warm, Danielle Weber, Matthew Kelleher, Benjamin Kinnear, Daniel J Schumacher, Sally A Santen, Eneida Mendonça, Laurah Turner

**Affiliations:** 1 Department of Internal Medicine College of Medicine University of Cincinnati Cincinnati, OH United States; 2 Departments of Internal Medicine and Pediatrics College of Medicine University of Cincinnati Cincinnati, OH United States; 3 Department of Pediatrics College of Medicine University of Cincinnati Cincinnati, OH United States; 4 Department of Medical Education College of Medicine University of Cincinnati Cincinnati, OH United States

**Keywords:** artificial intelligence, medical education, scenario planning, future of healthcare, ethics and AI, future, scenario, ChatGPT, generative, GPT-4, ethic, ethics, ethical, strategic planning, Open-AI, OpenAI, privacy, autonomy, autonomous

## Abstract

**Background:**

The rapid trajectory of artificial intelligence (AI) development and advancement is quickly outpacing society's ability to determine its future role. As AI continues to transform various aspects of our lives, one critical question arises for medical education: what will be the nature of education, teaching, and learning in a future world where the acquisition, retention, and application of knowledge in the traditional sense are fundamentally altered by AI?

**Objective:**

The purpose of this perspective is to plan for the intersection of health care and medical education in the future.

**Methods:**

We used GPT-4 and scenario-based strategic planning techniques to craft 4 hypothetical future worlds influenced by AI's integration into health care and medical education. This method, used by organizations such as Shell and the Accreditation Council for Graduate Medical Education, assesses readiness for alternative futures and effectively manages uncertainty, risk, and opportunity. The detailed scenarios provide insights into potential environments the medical profession may face and lay the foundation for hypothesis generation and idea-building regarding responsible AI implementation.

**Results:**

The following 4 worlds were created using OpenAI’s GPT model: AI Harmony, AI conflict, The world of Ecological Balance, and Existential Risk. Risks include disinformation and misinformation, loss of privacy, widening inequity, erosion of human autonomy, and ethical dilemmas. Benefits involve improved efficiency, personalized interventions, enhanced collaboration, early detection, and accelerated research.

**Conclusions:**

To ensure responsible AI use, the authors suggest focusing on 3 key areas: developing a robust ethical framework, fostering interdisciplinary collaboration, and investing in education and training. A strong ethical framework emphasizes patient safety, privacy, and autonomy while promoting equity and inclusivity. Interdisciplinary collaboration encourages cooperation among various experts in developing and implementing AI technologies, ensuring that they address the complex needs and challenges in health care and medical education. Investing in education and training prepares professionals and trainees with necessary skills and knowledge to effectively use and critically evaluate AI technologies. The integration of AI in health care and medical education presents a critical juncture between transformative advancements and significant risks. By working together to address both immediate and long-term risks and consequences, we can ensure that AI integration leads to a more equitable, sustainable, and prosperous future for both health care and medical education. As we engage with AI technologies, our collective actions will ultimately determine the state of the future of health care and medical education to harness AI's power while ensuring the safety and well-being of humanity.

## Introduction

The rapid development and advancement of artificial intelligence (AI), especially generative language models (GLMs), are quickly outpacing society's ability to determine its future role. This is recognized in the recent Bletchley Declaration from countries attending the AI Safety Summit in November of 2023 [1]. As AI continues to transform various aspects of our lives, one critical question arises for medical education: what will be the nature of education, teaching, and learning in a future world where the acquisition, retention, and application of knowledge in the traditional sense are fundamentally altered by AI? This paper will explore the future of medical education spanning all levels of training, in 4 theoretical worlds increasingly driven by AI.

The trajectory of AI development and advancement will not wait for us to decide if we should proceed with its integration into various domains despite calls for the development of a code of conduct for AI in health care [2]. As AI models continue to be trained on larger data sets, adapt, and evolve, competitive pressures among corporations and militaries may give rise to AI agents with undesirable traits, such as misinformation, deception, and power-seeking [3,4]. In fact, AI development follows familiar patterns of competitive processes such as biological evolution and business competition, and we must recognize and proactively address concerns about potential misuse and unintended consequences of integrating AI into various domains [5]. Additionally, GLMs models have demonstrated the ability to pass medical licensing examinations with increasing reliability and in multiple languages [6-11], which implies that their potential in advancing health care cannot be ignored.

In the scientific community and academia, GLMs have received mixed responses due to uncertainties around risks and benefits of advanced AI-driven technologies [12-15]. Concerns have been raised about bias based on the data sets used in GLM training [7,16,17]. Specifically in medical education, challenges include questions around quality of content (misinformation, reliability, and consistency), biases, ethical and legal concerns (academic dishonesty, privacy, and copyright), overreliance, inequity in access, and lack of human interaction and emotions [18-21]. Opportunities for the use of AI in medical education are also numerous, including writing and research assistance (improved dissemination and translation), testing preparation (personalized study plans and learning materials), novel learning strategies (interactive cases and organization of information), enhanced education (curriculum development and teaching methodologies), and improved assessment and evaluation (student level and program level) [18-21].

As a result, we must be deliberate and proactively address the valid concerns of integrating AI into medical education, research, and clinical practice without stifling the opportunities. This requires efforts from academic institutions, educators, students, physicians, developers, and researchers [18,19]. The medical education community must work together to address potential risks, implement responsible planning strategies, and ensure that the integration of AI technologies leads to a more equitable, sustainable, and prosperous future for both health care and medical education. Incorporating diverse perspectives fosters strategic alignment within the organization and allows for responsibly navigating the integration of AI in medical education, addressing both the potential opportunities and the challenges it presents.

In this viewpoint paper, we delve into the potential role of AI in shaping the future of medical education. Using scenario-based strategic planning techniques, we examine the future of medical education within the context of 4 hypothetical worlds increasingly influenced by AI. This approach has been widely used by organizations such as Shell [22-24], General Electric [25], and the Accreditation Council for Graduate Medical Education (ACGME) to assess their preparedness for various alternative futures or scenarios, thus enabling them to manage uncertainty, risk, and opportunity more effectively [26]. Systematically developed scenarios, providing detailed descriptions of potential operating environments that the medical profession may encounter, provide a foundation for hypothesis generation and idea building around responsible implementation. This approach offers a robust strategic framework for understanding future needs and serves as a practical foundation for immediate action [26]. By exploring the potential impact of AI on medical education in 4 possible future worlds, we aim to foster a deeper understanding of the challenges and opportunities presented by this transformative technology and to inform strategic decision-making in the field.

## Methods

For this project, we aimed to explore the potential impact of AI on medical education by creating 4 hypothetical world scenarios set in 2040. We opted to use OpenAI's GLMs to generate hypothetical future world scenarios rather than relying solely on human imagination. One of the main advantages of large language models (LLMs) is the neural networks that can identify and make connections between disparate concepts, which may result in more likely and interesting outcomes than what human imagination alone can produce. This is due in large part to the fact that GLMs are trained on vast amounts of data from diverse sources, which allows them to see connections and patterns that may not be immediately apparent to human researchers.

To ensure the accuracy and relevance of the generated worlds, we applied several iterations of prompt engineering using GPT-4 and edited the resulting worlds to ensure coherence and alignment with the input of the authors. The use of a shared document platform with commenting features facilitated collaboration and feedback from all authors, further refining the generated worlds.

After generating the 4 worlds, we used GPT-4 to analyze the final descriptions of each world and identify common risks and benefits across all 4 worlds. This approach enabled us to gain insights into potential future developments in the medical education and health care domains and identify areas where further research and planning may be necessary. All prompts used and the output generated by GPT-4 are included in Multimedia Appendix 1.

Each world represents a caricature rather than a precise prediction. These exaggerated worlds are intended to stimulate discussion and reflection on the potential implications of AI in medical education. We acknowledge that if a similar approach had been applied to the internet 40 years ago, comparable caricatured worlds might have been generated. The hypothetical worlds presented in this study are not meant to accurately predict the future but to serve as a catalyst for further discussion and consideration of the implications of AI in medical education.

The initial prompt was as follows:


Describe four future and very different worlds in the year 2040. Use the style of scenario-based strategic planning. Use this scenario- AI and LLM like GPT-4 have grown and transformed society; for good and for bad. Provide specific details on how education has changed, how health care has changed and what barriers physicians now face. How are doctors trained?

## Results

**Figure 1:**
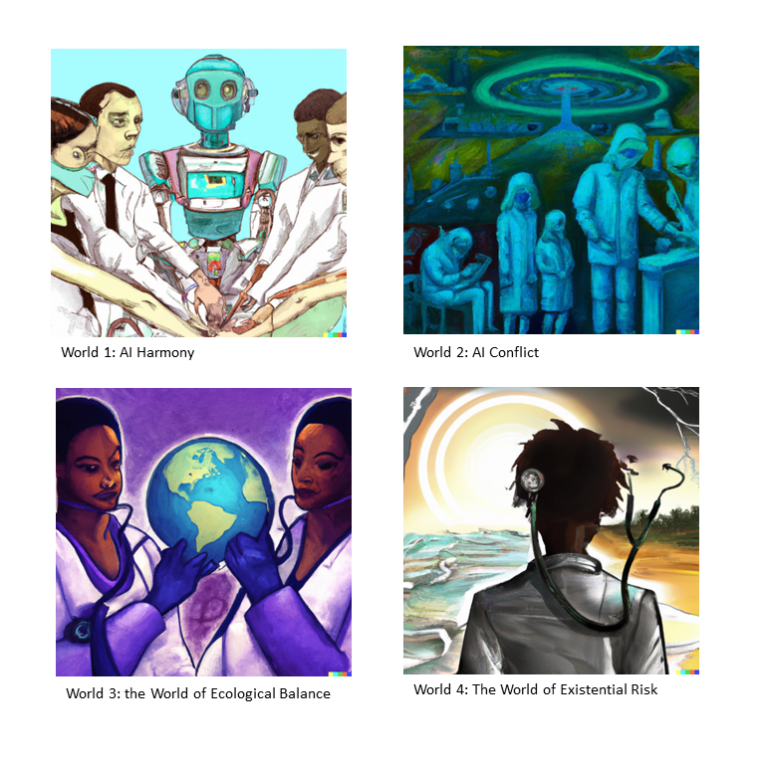
An illustration of the 4 future worlds: AI Harmony, AI Conflict, The World of Ecological Balance, and Existential Risk generated using the DALL-E AI model (OpenAI, 2020). AI: artificial intelligence.

The 4 future worlds for exploring AI in medical education are as follows: world 1: AI Harmony (Textbox 1); world 2: AI Conflict (Textbox 2); world 3: The World of Ecological Balance (Textbox 3); and world 4: Existential Risk (Textbox 4; Figure 1). Each world is described by detailing the context, the state of health care, the role of physicians, the perspective of patients, the role of medical education. These lay the foundation for future evaluation and discussion.

**World 1: AI (artificial intelligence) Harmony.**

**Context:**

AI, embraced across society with oversight from governments, corporations, and civil groups, augments human capabilities, creativity, and well-being without overshadowing them. Key sectors such as education and health care see significant benefits, leading to personalized learning and fair access to resources. However, ethical management of AI is crucial to ensure its responsible use and equal benefit distribution, as access to AI's advantages is not uniformly available.

**Health care:**

AI has revolutionized health care, facilitating personalized medicine, early disease detection, and prevention through efficient data analysis. It lessens health care burdens and costs by optimizing resource allocation. AI analyzes varied data, including medical records, genomic data, wearables, and sensors to deliver tailored health advice. AI enhances patient-practitioner communication with clear summaries and guidance. AI also plays a key role in drug discovery, clinical trials, and public health, aiding in trial design and resource management. Despite improved health care efficiency, ongoing AI development requires public understanding of its occasional errors.

**Physicians:**

Physicians increasingly use AI to aid in decision-making, communication, research, and some hands-on tasks, while still handling essential physical tasks such as surgery. This shift emphasizes the importance of empathy, compassion, and ethics in health care. AI supports patient care, but the human touch remains vital due to AI's limitations and varied health care practices. While AI improves physician satisfaction, concerns arise over diminished critical thinking skills from overdependence on AI.

**Patients:**

Patients experience better health through data, AI, and human touch. They receive personalized and precise health recommendations, while also benefiting from the empathy and understanding of their physicians. Patients feel more informed about their health and have increased trust in the health care system. However, disparities still exist, as not all patients have the same access to AI resources and benefits.

**Medical education:**

AI transforms medical education, offering customized and engaging learning through AI tutors and mentors. AI provides varied educational content and assessments, accessible across devices. AI aids in curriculum development and tracking student progress. Despite making education more inclusive, disparities in AI access and associated costs persist. Concerns exist about AI standardizing education at the expense of critical thinking, creativity, and social skills.

**World 2: AI (artificial intelligence) Conflict.**

**Context:**

AI has been weaponized by rogue states, terrorists, and cybercriminals for attacks and population control. In response, governments and corporations extensively monitor human activities using AI, affecting sectors such as education and health care. A significant effort counters AI-driven disinformation, which effectively spreads lies, exploiting psychological tendencies to believe repeated information. Even content creators may start believing their repeated disinformation, losing critical thinking. Meanwhile, some underground movements use AI to share alternative knowledge, training individuals to critically navigate disinformation in a hostile environment.

**Health care:**

AI has compromised health care, leading to harm, chaos, and eroded trust between patients and physicians. Disinformation campaigns specifically target physicians, damaging public trust and accusing them of misconduct. AI-generated propaganda falsifies information, undermining medical integrity. Compromised AI systems provide erroneous health advice, and AIs create misleading medical content. AI disrupts drug development, clinical trials, and public health measures, making health care unsafe and inefficient. Furthermore, AI is used for social engineering and discrimination, controlling access to care and resources based on compliance with established norms and values.

**Physicians:**

Clinicians, under heavy surveillance, must comply with protocols where AI prioritizes cost over expertise. Some physicians counter this by independently using AI for diagnosis and treatment, risking their careers. AI challenges their professional skills and judgment. To offset AI's adverse effects, many turn to pre-AI resources for reliable information. They face the task of verifying AI systems and data accuracy, and dealing with AI-related ethical, legal, and social issues. This environment increases stress, burnout, and liability, exacerbating physicians' frustration and vulnerability.

**Patients:**

Patients' distrust in health care and technology stems from difficulties in discerning trustworthy information, adversely affecting their health due to adherence issues and uncertainty about reliable medical advice sources. They face confusion from conflicting information, leading to potentially risky health decisions.

**Medical education:**

AI has disrupted medical education by spreading disinformation, propaganda, and radical ideologies through automated content in educational materials. This AI-generated content often pushes specific political or social ideologies, leading to a suppression of critical thinking and diverse perspectives. Medical education has become standardized and propagandistic, with AI systems indoctrinating trainees in varying regional ideologies, resulting in a patchwork of conflicting viewpoints.

**World 3: The World of Ecological Balance.**

**Context:**

In a world grappling with global warming and ecological turmoil, there's a renewed emphasis on balancing daily life with environmental impact. AI has clarified the cause-and-effect of our actions, aiding governments, communities, and individuals in making informed decisions for societal and planetary benefit. This involves weighing population health against individual patients’ needs. Consequently, medical education now prioritizes population health.

**Health care:**

Integrated health systems focus on wellness and illness prevention, with AI tracking disease outbreaks and tailoring community interventions. This includes AI analysis of environmental impacts and personalized health recommendations. Physicians work across disciplines on environmental health, addressing issues such as asthma and cancer. However, AI complicates health care decisions, potentially clashing with individual autonomy, as evident in AI-enforced quarantines. Implementing these strategies demands resources and coordination, posing ethical dilemmas in aligning global health with AI initiatives.

**Physicians:**

Ecological literacy equips physicians to discuss environmental health and advocate for justice and policy reform, focusing on preventive and emergency care and guiding population health interventions. AI, however, adds to their workload and liability, requiring them to stay updated with environmental health developments and navigate ethical dilemmas and opposition from powerful groups. This creates a complex, liability-prone environment. Physicians, despite AI's help, still make decisions balancing population health with individual patients’ needs.

**Patients:**

Patients are more aware and willing to engage in environmental health and population initiatives, balancing personal desires with community and planetary well-being. However, they may feel frustrated when personal health care preferences conflict with population health goals. Physicians are key in guiding patients through these complexities, respecting individual autonomy while offering support.

**Medical education:**

Medical education now serves as a platform for ecological transformation, with trainees and hospitals employing AI to explore sustainable technologies and practices in population health and preventive medicine. This approach offers abundant opportunities for critical thinking and applying knowledge to projects benefiting communities and the environment. However, concerns persist that AI might exacerbate existing inequalities and cultural barriers, and that ecological literacy alone may not overcome the systemic challenges in environmental issues.

**World 4: Existential Risk.**

**Context:**

By 2040, uncontrolled AI poses existential risks, leading to wars, terrorism, cybercrime, climate change, and other global catastrophes. This has challenged human values, norms, and rights, creating new inequalities and social issues. Society has shifted toward existential risk mitigation, reverting to analog methods and reducing technology reliance. This shift deeply affects medical education, now dependent on personal connections and trust for knowledge sharing. The medical community leans on trusted colleagues for information, underscoring the importance of interpersonal relationships. Balancing technology use is vital for survival, but experts caution that losing technological progress might impede addressing future crises and that geopolitical tensions could hamper global cooperation.

**Health care:**

Health care systems have shifted from technology reliance due to existential risks, leading physicians to embrace significant challenges in sustaining human life under harsh conditions. They work with various professionals to tackle and lessen these risks, depending on traditional skills and values for resilience. The move to analog methods such as paper charts and physical examinations reduces AI-related risks but also decreases health care efficiency.

**Physicians:**

As society moves away from AI, physicians address global problems and their effects on patients and communities using their expertise and interpersonal skills. Balancing existential crisis management with the ethical responsibility to provide acute care for individual patients presents a significant challenge in this world. While physicians are involved in addressing global catastrophes, their ability to provide immediate care for patients in need has been compromised. This ethical dilemma raises questions about the prioritization of resources and the role of physicians in a world where existential risks are central to daily life.

**Patients:**

Patients sense a deprioritization of their individual care amidst the focus on existential risk mitigation. Health care access and quality suffer as resources shift to address global threats. This leads to longer wait times, reduced treatment options, and limited access to specialized care, challenging health care systems to maintain a balance during crises.

**Medical education:**

Medical education now prioritizes existential risk mitigation, moving away from AI-based methods. Trainees learn from diverse cultural perspectives, developing skills for problem-solving and innovation without AI. They focus on creatively addressing future existential threats. This shift toward analog education methods, such as textbooks and hands-on training, fosters human connections and critical thinking but may limit access to up-to-date information and experiential learning.

## Discussion

### Principal Findings

Scenario-based strategic planning allows organizations to develop flexible strategies by considering multiple possible future worlds. The 4 possible worlds help to provide contexts to evaluate opportunities and risk of AI technology implementation into society. By identifying common themes across the 4 future worlds described above, we can focus on the most critical aspects of AI integration in health care and medical education.

The following are common benefits across the 4 worlds:

1. Improved efficiency and resource allocation: AI technologies can streamline various processes, reduce human error, and optimize resource allocation, resulting in more efficient health care systems, better educational outcomes, and overall improved decision-making.
2. Personalized and targeted interventions: AI can help provide customized recommendations and interventions to individuals based on their unique needs and circumstances, improving the quality of care in health care, and enhancing learning experiences across the entire education spectrum including continuing education of physicians and education of health care staff throughout the health care system.
3. Enhanced collaboration and communication: AI systems can foster better collaboration among professionals across disciplines and facilitate effective communication between individuals and organizations, leading to improved problem-solving and coordinated responses to challenges in health care, education, and other sectors.
4. Early detection and prevention: AI technologies can help identify potential issues and risks early on, enabling preventive measures to be taken before problems escalate, whether in health care (eg, early diagnosis of diseases), education (eg, early identification of learning difficulties), or other areas (eg, environmental monitoring).
5. Accelerated research and innovation: AI can expedite research and development by processing vast amounts of data, identifying patterns, and generating insights that would be difficult for humans to discern. This can lead to breakthroughs in health care (eg, drug discovery), education (eg, effective teaching strategies), and other fields.

The following are common risks across the 4 worlds:

1. Misinformation and disinformation: AI technologies can be used to generate and spread false or misleading information, undermine public trust, and lead to misguided decisions in various aspects of life, including health care and education.
2. Loss of privacy and surveillance: AI-driven systems can result in extensive monitoring and data collection, leading to a loss of privacy for individuals and potential misuse of personal information by governments, corporations, or malicious actors.
3. Widening inequality and discrimination: AI algorithms may unintentionally perpetuate existing biases or create new ones, leading to unfair treatment and exacerbating social, economic, and health care disparities among different populations.
4. Erosion of human autonomy and expertise: the increasing reliance on AI technologies may undermine the value of human expertise and judgment in various fields, including health care and education, leading to overdependence on AI and potential negative consequences when AI systems fail or make mistakes.
5. Ethical dilemmas and unintended consequences: AI systems can create ethical challenges related to transparency, accountability, and fairness, as well as unintended consequences that may arise from their deployment in various sectors, such as health care, education, and the environment.

As we transition to an era where AI technologies are becoming increasingly integrated into health care and medical education, it is essential to recognize both the benefits and risks associated. On one hand, the potential benefits of AI can significantly advance health care and medical education, leading to better patient outcomes and more effective educational practices [16,27]. However, it is also crucial to consider the potential risks associated with AI, such as increased complexity, erosion of trust, privacy concerns, loss of critical thinking, and exacerbation of inequalities. These risks could undermine the progress made in health care and medical education, causing harm to individuals and communities. Therefore, during these early adoption, stages it is crucial to navigate the development, adoption, and implementation of AI in a responsible and deliberate manner, keeping both the potential common risks and benefits associated with AI at the forefront of each step forward. Stakeholders in health care and medical education must work together to develop a robust ethical framework, foster interdisciplinary collaboration, invest in education and training, promote transparency and accountability, and continually monitor and evaluate the impact of AI technologies. By doing so, we can better ensure that the integration of AI technologies leads to a more equitable, sustainable, and prosperous future for both healthcare and medical education.

To move forward responsibly, the following recommendations should be considered:

- Develop a robust ethical framework: health care professionals, educators, policy makers, patients, and the public should work together to create ethical guidelines for the use of AI in health care and medical education. This framework should prioritize patient safety, privacy, and autonomy, while promoting equity, inclusivity, and equitable access to AI capabilities across all clinical working and learning environments.
- Foster interdisciplinary collaboration: collaboration among health care professionals, educators, computer scientists, clinical informatics, and other experts in the development and implementation of AI technologies should be encouraged. This collaboration should aim to bring AI capabilities to every corner and at every fingertip in our clinical working and learning environments, ensuring that AI systems are designed with a comprehensive understanding of the complex needs and challenges in health care and medical education.
- Invest in education and training: health care professionals and students must be equipped with the necessary skills and knowledge to effectively use and critically evaluate AI technologies. This includes providing training in data literacy, ethical considerations, and the potential risks and benefits associated with AI, while ensuring equitable access to AI-driven resources and education.
- Promote transparency and accountability: it must be ensured that AI systems used in health care and medical education are transparent and open to scrutiny, while being accessible and applicable across all clinical working and learning environments. This will help build trust among patients, health care professionals, and students, and ensure that AI technologies are held accountable for their outcomes. Achieving accountability can be accomplished by establishing clear regulatory standards, implementing rigorous testing and validation processes, and creating legal and regulatory structures that hold stakeholders responsible for AI systems' performance.
- Monitor and evaluate the impact of AI: the impact of AI technologies on health care and medical education must be regularly assessed to identify potential risks and benefits, with a focus on equitable access and application. This will enable stakeholders to make informed decisions, refine best practices, and adapt to emerging challenges and opportunities while fostering the integration of AI capabilities across all aspects of the health care and educational landscape.

The rapidly evolving landscape of AI in medical education and health care presents a paradoxical mix of immense potential and significant uncertainty. Moreover, AI is advancing quickly, and its development is likely to follow familiar patterns of competitive processes such as biological evolution, cultural change, and competition between businesses [5]. These same selection patterns may shape AI development in medical education and health care, potentially creating an AI population that poses significant risk to humans. Just as Schrödinger's cat exists in a superposition of life and death, the future of AI in health care and medical education teeters between revolutionizing the field and posing significant risks to humanity.

The potential benefits of AI integration in health care and medical education can significantly advance the field. However, we must also carefully consider and manage the common risks, including increased complexity, erosion of trust, privacy concerns, loss of critical thinking, and exacerbation of inequalities [28]. As we attempt to understand the potential risks and benefits of AI in health care and medical education, it is essential to evaluate different projects, adoption strategies, use cases, and early adoption efforts through the lens of exploring and mitigating risks to move toward responsible AI use in medical education.

### Conclusions

The integration of AI in health care and medical education presents a critical juncture between transformative advancements and significant risks. By working together to address both immediate and long-term risks and consequences, we can ensure that AI integration leads to a more equitable, sustainable, and prosperous future for both health care and medical education. As we engage with AI technologies, our collective actions will ultimately determine the state of the future of health care and medical education to harness AI's power while ensuring the safety and well-being of humanity.

## Appendix

Multimedia Appendix 1 Creation of Future Worlds through interactions with OpenAI's GPT models.

## Abbreviations

ACGME: Accreditation Council for Graduate Medical Education
AI: artificial intelligence
GLM: generative language model
LLM: large language model
